# Image-Guided Musculoskeletal Interventional Radiology in the Personalised Management of Musculoskeletal Tumours

**DOI:** 10.3390/jpm14121167

**Published:** 2024-12-21

**Authors:** Hasaam Uldin, Ibrahim Kanbour, Anish Patel, Rajesh Botchu

**Affiliations:** Royal Orthopedic Hospital, Birmingham B31 2AP, UK; hasaam.uldin@nhs.net (H.U.); anish.patel4@nhs.net (A.P.)

**Keywords:** radiology, interventional, orthopedics, tertiary care centres, tertiary healthcare, patient care, radiologists, neoplasms

## Abstract

Musculoskeletal image-guided interventional radiology plays a key role in diagnosing and treating a range of conditions. Recent advances have yielded a wide variety of procedures that can be applied selectively and enable the personalisation of patient care. This review aims to outline the indications, applications, and techniques of subspecialist musculoskeletal oncology interventional procedures that were used at our tertiary referral centre with a focus on how these may be used to personalise patient management. The applications of a range of diagnostic and therapeutic image-guided interventional procedures including different methods of bone and soft tissue sampling, ablation, and augmentation procedures across different types of patients and pathologies are reviewed. To supplement the reviewed literature, we included our own experience and radiology images retrospectively collected from our Picture Archiving and Communication System (PACS). We demonstrate how the range of musculoskeletal image-guided interventions provide flexibility in the diagnosis and management of different tumours across different patient populations. This study provides the musculoskeletal interventional radiologist with insight into how to appropriately utlilise different techniques to optimise the diagnosis, treatment and palliation of tumours.

## 1. Introduction

Musculoskeletal oncology is a large and diverse field of growing complexity and scientific development with rapidly emerging medical and surgical developments [1]. Radiology is a key facet of this paradigm shift, in large part thanks to the rapid development of musculoskeletal diagnostic and therapeutic interventional radiology (IR) [2].

Radiology in general has advanced through improvements in radiography, ultrasound, fluoroscopy, Computed Tomography (CT), and Magnetic Resonance Imaging (MRI). These have accelerated the role radiology plays in diagnosis and follow-up of soft tissue and bone tumours. Musculoskeletal IR has more directly benefited from a host of other technological innovations by enabling far more options and flexibility in the options available for minimally invasive procedures.

Musculoskeletal IR entails two main types of procedures. The first are diagnostic procedures, which are used in conjunction with a clinical and radiological assessment to better characterise lesions and subsequently optimise the wider patient management [3,4]. In the context of bone and soft tissue tumours, such procedures predominantly involve biopsy to facilitate a positive diagnosis. Therapeutic options are generally more wide-ranging and we will discuss various types of ablation and augmentation procedures. Treatment aims can be curative or palliative and minimally invasive intervention can play a valuable role in optimising patient care in both types of cases, either alone or in conjunction with other treatments [5].

This review covers the current state of musculoskeletal interventional procedures within the setting of our tertiary tumour centre. The focus is on the principles behind the different techniques used and the flexible application of these to manage a range of conditions.

## 2. Methods

Ultrasound and CT interventional radiology lists at our tertiary orthopaedic oncology centre take place most days of the week. A retrospective search of these lists for procedures performed for oncological indications was carried out using the Radiology Information System (RIS). The indications and techniques of the included procedures were considered. Radiology images were collected from our Picture Archiving and Communication System (PACS).

To support our analysis of the procedures at our centre and contextualise findings within the context of the wider literature, a literature search was carried out in Medline using the terms ‘musculoskeletal’, ‘oncology’, ‘biopsy’, ‘intervention’, and ‘radiology’. The relevant existing literature was reviewed and forms part of the discussion of this review.

## 3. Review and Discussion

### 3.1. General Considerations

Preprocedural planning is perhaps the most crucial aspect of interventional radiology in general and musculoskeletal interventions are no different [3]. Meticulous patient selection, review of existing imaging, and patient preparation are needed before the patient even reaches the interventional radiology department. From our experience, optimisation of these parameters can make even the most difficult procedures proceed smoothly. Conversely, their neglect can be very detrimental and risks complications even in relatively straightforward procedures.

Thorough planning also contextualises any procedures within the wider perspective of patient care. Different patients will have different needs leading to different treatment objectives. Every patient at our institution is, therefore, discussed at a multidisciplinary team (MDT) meeting to ascertain the aims of treatment and thus select the most appropriate procedure, if any. As discussed later in this text, radiological interventions can play a role in both definitive palliative and curative management as well as adjunctly support other treatments such as surgery and medical therapies [5].

Imaging plays a crucial role in narrowing the diagnosis and in selecting patients for biopsy [6]. A wide range of modalities are utilised including radiography, ultrasound, MRI, CT, and nuclear medicine studies. Although many soft tissue and bone lesions are difficult to fully assess on imaging, there are numerous ‘do not touch’ entities where the imaging characteristics are so clear that a biopsy or intervention would be of no additional benefit and is, therefore, obviated. At our institution, such cases are still discussed with the MDT to gain input from the clinical team before a decision is made not to actively treat.

Imaging also plays an invaluable role in planning percutaneous radiological procedures as well as oncological and surgical treatment [3,6]. The various modalities can help to plan approaches, make equipment choices, and monitor complications. Specific examples of this are discussed below.

### 3.2. Patient Discussion

It is important to remember the patient is at the centre of what is usually a complex care pathway which each clinician must help to navigate. For any musculoskeletal IR procedure, an explanation of the objectives, techniques, and role in overall care must be given at a prior date. This is typically carried out by the oncology team in our institution and forms the first part of the consenting process. On the day of the procedure, the patient meets with the performing interventional radiologist for a detailed discussion on the aims, methods, and risks of the procedure as well as any alternative options, thus completing the consent process. Patients will often raise concerns and queries about technical aspects of the anaesthetic and radiological processes which should be addressed in a comprehensive yet understandable manner.

Most cases at our institution are performed as day cases with patients arriving early in the morning for preprocedural checks and processes. Overnight stays may be necessary for more complex procedures or those with a higher risk of complications.

### 3.3. Procedure Setting

The bulk of intervention work at our institution is performed in a CT scanner room with an ultrasound machine also readily available. Patients are brought from the day case unit into an adjacent waiting recovery area where final preprocedural checks are carried out. Experienced interventional staff including radiographers, nursing, and anaesthetic staff who are familiar with the procedures performed are important to the smooth running and optimisation of patient experience for any procedure.

### 3.4. Diagnostic Procedures

As well as providing diagnostic information in and of itself, imaging is also a means to obtain tissue samples where histopathological analysis is needed for both soft tissue and bony lesions [7]. Several scenarios require such sampling, the most obvious being in cases of clinical and radiological uncertainty as to whether a lesion is benign or malignant. Even where lesions are almost certainly malignant, tissue sampling is usually helpful to identify whether the lesion is a primary tumour or metastasis and detailed histopathological analysis can aid personalisation of subsequent medical oncological care. At our institution, it is only in a few cases that the risk of sampling outweighs any potential palliative or curative benefit from a biopsy.

Most tissue sampling at our institution is carried out under radiological guidance. The main exceptions are very superficial lesions which can be sampled percutaneously without imaging and the minority of lesions which would require en bloc resection regardless of their histological constitution.

#### 3.4.1. Soft Tissue Biopsy

Diagnostic image-guided procedures are a staple of interventional radiology seen in generalist and specialist settings. In the context of musculoskeletal IR, percutaneous core biopsies are the most common procedure. Different approaches are used, mainly dependent on the site, size and composition of the lesion in question.

The easiest lesions to sample are those composed of homogenous soft tissue and located subcutaneously and remote from any important soft tissue structures (e.g., nerves, vessels, tendons, etc.). These can sometimes be biopsied without any imaging whatsoever. However, many lesions will have a degree of heterogeneity or be located within or adjacent to vulnerable structures. These benefit from direct visualisation. Ultrasound is the modality of choice as it provides high-resolution images of superficial structures allowing real-time direct visualisation of the needle as it is placed into the lesion, avoiding adjacent vital structures.

For soft tissue lesions, which are much deeper (e.g., in larger patients or deeper tissues) or more awkward or harder to visualise on ultrasound (e.g., obscured behind bone), CT is an option. While this does not allow real-time visualisation and imparts a radiation dose, visualisation of any area of the body using this generally more completely illustrates the salient anatomy. At our institution, this is carried out by obtaining an initial unenhanced planning helical scan. The skin is then marked and local anaesthetic is administered. Axial slices are then obtained at the site of needle insertion as the local needle is advanced and subsequently exchanged with a biopsy system.

We employ a ‘CT fluoroscopy’ method to increase accuracy, speed, and patient safety [8]. Unlike the conventional method of entering and exiting the scanner when scanning needle and lesion position, this alternative method consists of staying in the scanning room with the interventional radiologist operating a foot pedal to acquire Partial-Angle CT (PACT) images. Reducing time moving in and out of the scanner means images are acquired quicker thus minimising the dwell time of any needles thereby reducing the risk of inadvertent motion and the need for repeat imaging, ultimately minimising the radiation dose to the patient [9]. However, there is a radiation risk to the operator which must be minimised by ensuring the use of protective lead aprons, thyroid shields, and glasses. The operator’s dose is further minimised by limiting the volume of scanning and standing on the detector (rather than the tube) side of the scanner where the dose is minimised [10]. Of course, care should also be taken to only have staff in the room at the time of scanning who must be present, in our institution this is typically only the operating radiologist with an assistant for parts of the procedure where necessary.

**Figure 1:**
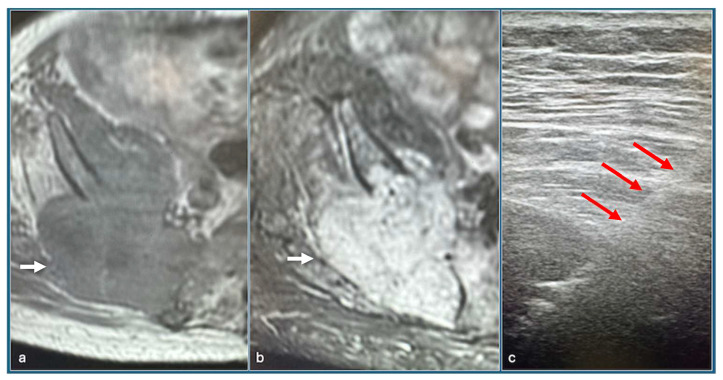
Axial T1 (**a**), STIR (**b**) shows tumour (arrow) involving right posterior ilium and Ultrasound-guided biopsy (**c**) showing needle (red arrows).

For soft tissue biopsies, we employ disposable 14-gauge spring-loaded core biopsy needles (Supercore), typically acquiring four to five samples (Figure 1). In superficial lesions, multiple passes can be made. For deeper lesions, a coaxial system is advised to allow easier biopsy with reduced discomfort and injury.

For all soft tissue and bone lesions apart from those which are immediately subcutaneous, knowledge of the surgical approach is crucial. Although there is some controversy, it is generally accepted that many tumours will seed along the biopsy tract necessitating its resection at the time of any surgery [11,12]. If the biopsy tract is remote to the surgical access, this necessitates widening the surgical field and can limit the extent of treatment as well as reconstruction options. It is, therefore, imperative to agree the biopsy approach with the surgical team prior to the procedure [3]. As a general rule for soft tissue lesions, one must aim not to involve additional compartments to the one involved and to avoid any major neurovascular structures [13].

#### 3.4.2. Bone Biopsy

Bone lesion biopsy is a similarly variable process. However, given that such lesions are almost invariably deep and typically have either a calcified component or lie behind a bony cortex, ultrasound is usually not a feasible option. Therefore, CT guidance is the workhorse of these procedures in our centre. As with soft tissue lesions, an initial planning scan is followed by local anaesthetic administration and then ‘CT fluoroscopy’ imaging as a percutaneous biopsy system is targeted to the lesion.

**Figure 2:**
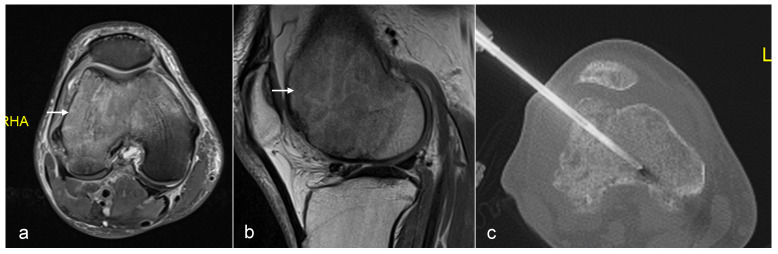
Axial T2fat suppressed (**a**), sagittal Proton density (**b**) showing tumour in the distal femur. Axial CT (**c**) showing biopsy needle in the distal femoral lesion.

**Figure 3:**
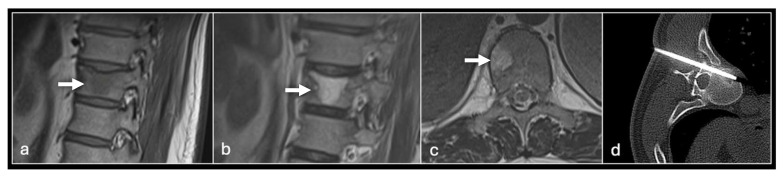
Sagittal T1 (**a**), T2 (**b**) and axial T2 (**c**) showing tumour (arrow) in the vertebral body and CT guided biopsy of the lesion (**d**).

We use two main needle systems. Most bone lesions can be sampled with a T-Lok eight-gauge system with an inner diamond-tipped stylet and outer cannula. This is inserted through the cortex overlying a bone lesion. Once in the lesion, the stylet is removed and the cannula is advanced to the deep wall of the lesion aiming to trap the core against normal bone on either side. A tray is then inserted through the cannula to acquire the sample. Both tray and cannula are withdrawn together and the sample is then deposited into the relevant histopathology or microbiology pots (Figure 2 and Figure 3). Typically, only one sample is needed unlike in a soft tissue biopsy.

While the T-Lok works well for lesions with relatively minimal overlying cortical bone, dense sclerotic bone can be difficult to traverse and requires a drill. Hand and battery-powered drills are available. We employ a Bonopty penetration set which includes a drill. The procedure is similar to with the T-Lok but the drill is inserted after the stylet is removed to penetrate sclerotic bone. The drill itself is taken out at the periphery of the lesion and the cannula is advanced. The radiologist must take care not to destroy the sample itself with the drill, which can easily be inadvertently achieved with small soft lesions within a sclerotic periphery. This is avoided by removing the drill just before entering the lesion itself.

Some bone lesions can consist of large soft-tissue components enclosed within cortical bone. These can be sampled by using a 10 cm × 8 G T-Lok system to gain access through the calcified component. The stylet is then exchanged with a 15 cm × 14 G Supercore biopsy needle (as used in soft tissue biopsy) passed through the cannula (functioning analogously to a coaxial needle) and multiple samples are taken.

Regardless of the type of lesion within the bone, bone biopsies can be technically challenging. A key reason for this is that needle systems are relatively heavy and can be difficult to stabilise under their weight whilst they are in soft tissue but not yet in bone. This can cause the needle to change position when it is released by the radiologist to allow scanning. This is particularly pronounced in the extremities due to the relatively thin, soft tissue and rounded structure. We use several techniques at our institution to adapt to this problem, including stabilisation with steristrips, anchoring against bones, and rolling sterile gauze or towels under the needle [14,15,16]. Once the needle enters into bone it is generally anchored enough to not move unless it is a particularly long or horizontally orientated needle.

#### 3.4.3. Aspiration

Purely cystic lesions—whether in bone or soft tissue—cannot be sampled using biopsy needles alone. Instead, aspiration is used to obtain the material for histopathological and microbiological analysis. Superficial lesions can be sampled with a 21 G 1.5″ ‘green’ needle under ultrasound guidance. Deeper lesions often require a Quincke-style 22 G needle up to 7″ long. Cystic lesions may be enclosed within the bone and can be accessed using a bone biopsy system with subsequent aspiration through the cannula.

### 3.5. Percutaneous Therapeutic Procedures

Indications for therapeutic musculoskeletal IR procedures fall into two broad categories. Some procedures are performed for definitive treatment, usually of small and isolated lesions that can be targeted and treated percutaneously. These percutaneous therapies have a role as they are minimally invasive and, therefore, generally better tolerated than the surgical alternatives [17,18].

Aside from curative intent, many treatments are carried out for palliative purposes in the context of widespread disease or frailty precluding definitive medical or surgical treatment. As discussed in more detail below, this can include treatment of symptomatic lesions or consolidation of pathological fractures via augmentation procedures. Whilst not ridding the patient of disease, the impact of reduced pain and disability on improving the quality of life achieved through a relatively tolerable procedure can be remarkable.

#### 3.5.1. Ablation

Percutaneous ablation is the destruction of abnormal tissues within bone or soft tissues using needles introduced under image guidance. As with diagnostic procedures, this is a minimally invasive alternative to other options such as surgery. A range of procedures lie under this umbrella but several general principles apply. Regarding indications, ablation can be a curative or adjuvant therapeutic option.

At our institution, ablation is almost always performed with CT guidance which optimises logistical efficiency and procedure planning but some types of ablation can be performed purely under ultrasound, for instance for Morton’s Neuroma [19,20]. As for other interventional procedures, imaging is reviewed and a path to the soft tissue is planned. The patient is placed on the scanner in the appropriate position and a radio-opaque grid is applied to the vicinity of the lesion. Following a planning scan, the skin is marked in one or more locations. Local anaesthetic is then administered to subcutaneous tissues. Access to the lesion is usually obtained using either bone or soft tissue biopsy systems (as above) which allows a sample of the lesion to be obtained before ablation. Once the biopsy needle is withdrawn, the cannula remains in situ and an ablation needle is passed through this.

We use two main categories of ablation at our institution—chemical and thermal (see Table 1). The former category largely entails sclerotherapy. From the latter, we mainly apply radiofrequency ablation and cryoablation with other types of energy-based ablation such as microwave, laser, and MRI-guided high-frequency ultrasound rarely used at our institution.

##### Sclerotherapy

Sclerotherapy is the injection of a sclerosant into a tumour to induce chemical ablation [21]. It is the primary form of chemical ablation for musculoskeletal tumours at our centre. It is mainly used for the treatment of cystic entities—most commonly aneurysmal bone cysts (ABC) but also venous malformations and giant cell tumours (GCTs)—as an alternative to surgical options [22]. It can also be used as an adjuvant treatment for locally aggressive GCTs and cystic malignant tumours but surgery en bloc remains the treatment of choice for these lesions.

A wide range of injectants are used including polidocanol, ethanol, and warm Ringer’s lactate (Hartmann’s) solution [13,15,16,17,23,24]. At our centre and others, doxycycline is agitated with an equal volume of air and used for injection [25]. This antibiotic has shown safety and efficacy in suppressing tumour growth and stimulating bone formation, thus rendering it a useful agent for sclerotherapy in both appendicular and axial lesions [14,18,19,25,26,27].

**Figure 4:**
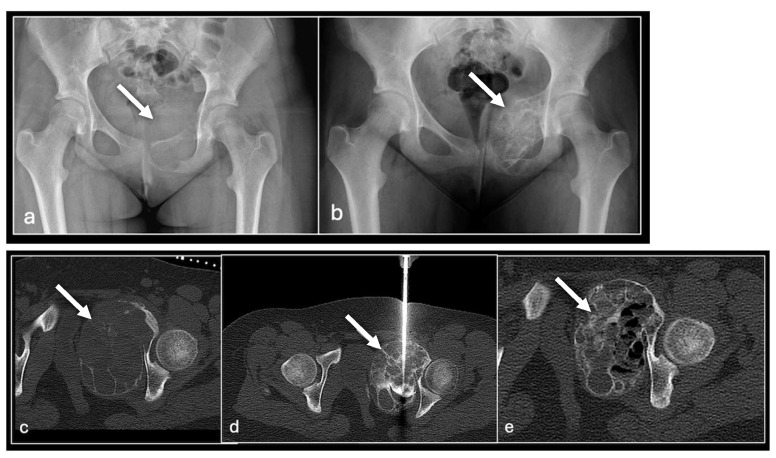
AP radiograph of pelvis (**a**) showing destructive lesion of left pubis and superior pubic ramus (arrow) with marked consolidation post three sessions of sclerotherapy (**b**). Axial CT imaging pre sclerotherapy (**c**), intraprocedural (**d**), and following three sessions of sclerotherapy (**e**) to the lesion (arrow).

**Figure 5:**
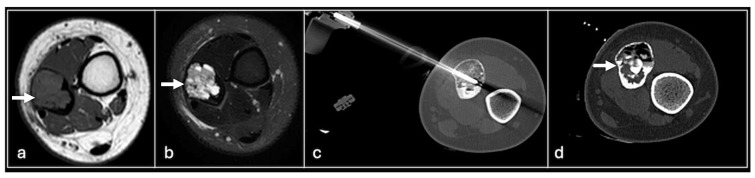
Axial T1 (**a**), STIR (**b**) showing aneurysmal bone cyst of fibula (arrow) treated with sclerotherapy under CT guidance (**c**,**d**).

The cyst is typically accessed using an 11 G bone biopsy needle. If the lesion is multiloculated, the needle is agitated to break up the septations to facilitate communication and its contents are aspirated. The position is confirmed with the injection of iodinated contrast which also has the benefit of confirming no intravascular communication or leak into other adjacent structures is present as also described in the existing literature [28,29]. If the lesion cannot be completely opacified, further punctures may be required. Once satisfactorily opacified, the sclerosant is injected and subsequently aspirated to minimise the risk of leakage. This allows the lesion to consolidate or involute over successive procedures (Figure 4 and Figure 5).

Sclerotherapy advantageously does not suffer from the risks to adjacent tissues and skin seen with the thermoablation techniques described above. Complication rates are low but sclerosant leakage or injection into vascular structures is a potential risk which can lead to embolism and tissue necrosis. Perhaps the biggest drawback is that repeat procedures are frequently necessary, particularly for larger lesions resulting in substantial time and resource allocation [30,31,32].

##### Thermoablation

In thermoablation, the aim is to rapidly change cell temperature to induce necrosis or coagulation in tumour cells while preserving the adjacent normal cells, the latter of which are typically more resistant to temperature change [33]. The ablated area then involutes and is replaced with soft tissue or bone over subsequent weeks to months. Augmentation procedures (see below) can be used in larger ablated areas to maintain structural integrity during the healing process [34].

Radiofrequency ablation (RFA), microwave ablation (MWA), interstitial laser ablation (ILA), and high-frequency ultrasound (HIFU) all apply ablative heat energy in different ways. Cryoablation instead uses ice balls to destroy tissue.

• Radiofrequency Ablation

Radiofrequency ablation has been used for over a century in a range of non-musculoskeletal and non-oncological settings [35,36]. Its application to tumour ablation has increased in recent years. There are several types, with our institution using pulsed RFA.

This technique is particularly used as a first-line curative option in osteoid osteomas and osteoblastomas. It is also effective in the cure of chondroblastoma [37,38]. As a palliative option, it has been used for painful bony and soft tissue metastases as well as myeloma and can be combined with cement augmentation to restore function [36,39,40].

Radiofrequency ablation uses temperatures of up to 95 °C applied over several minutes to induce tumour cell coagulation necrosis. Technically, this is achieved by positioning an RFA needle (the cathode) into the lesion using a similar approach to a bone biopsy (i.e., with a coaxial biopsy needle system including a drill) and applying a grounding pad to the patient’s thigh (the anode). Most treated lesions are surrounded by cortical bone, thus insulating adjacent structures from the hot needle tip and effectively heating the tumour. Before introducing the RFA needle, a biopsy is typically taken to confirm the suspected diagnosis.

**Figure 6:**
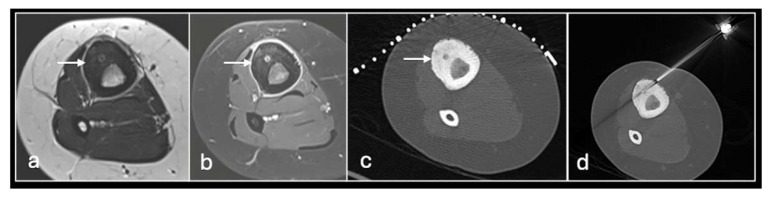
Axial T1 (**a**), STIR (**b**), CT (**c**) showing osteoid osteoma of tibia (arrow) treated with radiofrequency ablation (**d**).

**Figure 7:**
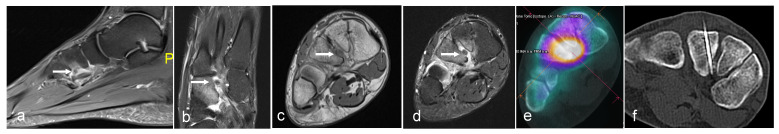
Sagittal (**a**) and axial (**b**) T2 fat-suppressed, coronal PD (**c**) and PD fat-saturated (**d**), SPECT CT (**e**) all showing osteoid osteoma of the lateral cuneiform (white arrows) treated with radiofrequency ablation (**f**).

**Figure 8:**
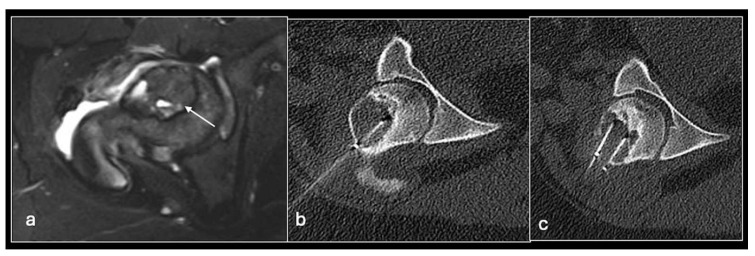
Axial STIR (**a**) showing femoral head chondroblastoma (arrow) treated with CT-guided radiofrequency ablation with multiple needles (**b**,**c**).

**Figure 9:**
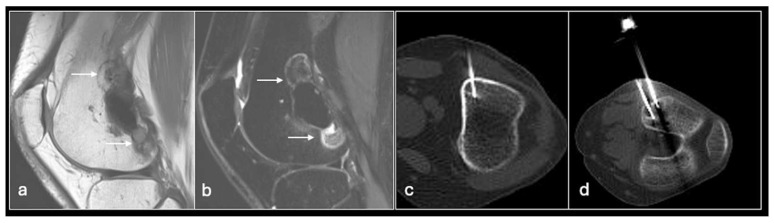
Sagittal T1 (**a**), STIR (**b**) showing recurrence of GCT cranial and caudal (arrows) to prior cementation subsequently treated with radiofrequency ablation (**c**,**d**).

**Figure 10:**
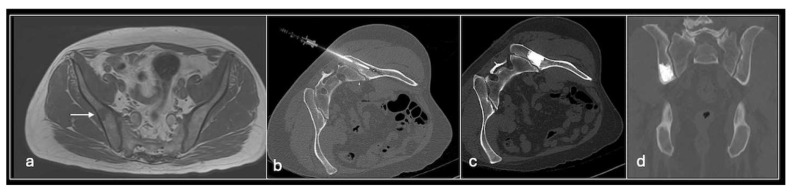
Axial T1 (**a**) showing metastasis in right ilium (arrow) (**b**) treated with radiofrequency ablation. Post-cementoplasty axial (**c**) and coronal (**d**) CT post-cementoplasty.

**Figure 11:**
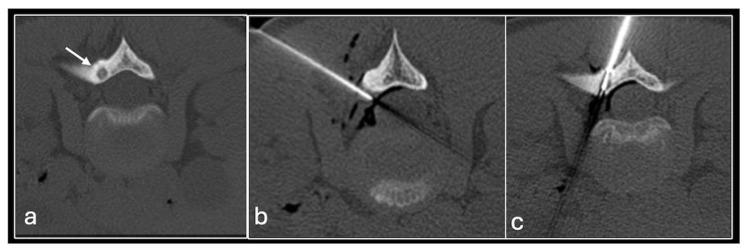
Axial CT showing osteoid osteoma of lumbar spine (arrow) (**a**) treated with radiofrequency ablation with neuroprotection using air (**b**,**c**).

As well as bearing in mind the ‘standard’ complications associated with percutaneous procedures involving bone such as fracture, infection, pain, and injury to neurovascular structures, RFA also can cause thermal injuries perilesional soft tissue as well as significant skin burns at the needle and grounding pad sites. Techniques such as injection of fluid or CO_2_ to separate structures or thermal monitoring of adjacent tissues can minimise the risk of injury (Figure 6, Figure 7, Figure 8, Figure 9, Figure 10 and Figure 11).

• Microwave Ablation

Microwave ablation (MWA) is another heat-based method whereby antennae are inserted into the tumour and an electromagnetic field is used to generate the heat. The advantages to this method over RFA include faster induction of coagulation necrosis, reduced heat loss, and less variably inherent resistance [41,42]. These features allow deeper extension and a larger ablation zone. Like RFA, MWA risks burning the ablation site but does not use a grounding pad and, therefore, does not result in skin burns here [43].

In the liver, MWA shows a more consistent and faster response than RFA and allows the use of multiple probes. These factors favour its suitability for larger tumours and those in close proximity to neurovascular structures [44]. The literature delineating the role of MWA in musculoskeletal tumour treatment is relatively sparse but several successful series have found MWA is an effective curative option for osteoid osteomas, perhaps superior to RFA [41,43].

• Laser Ablation

Laser ablation is also known as Interstitial Laser Ablation (ILA) or Laser Interstitial Thermal Therapy (LITT). The underpinning principle is the delivery of infrared light through optical fibres to generate head and induce coagulation necrosis [45]. This is a relatively new method first reported in 1997 but key potential benefits over MWA and RFA are more precise and predictable energy delivery into the lesion as well as MRI compatibility [46,47]. Efficacy was demonstrated in small benign tumours (such as osteoid osteomas and vascular malformations) as well as malignant tumours outside of bone but no clinical applications for malignant lesions are present in the existing literature [48,49].

• Cryoablation

**Figure 12:**
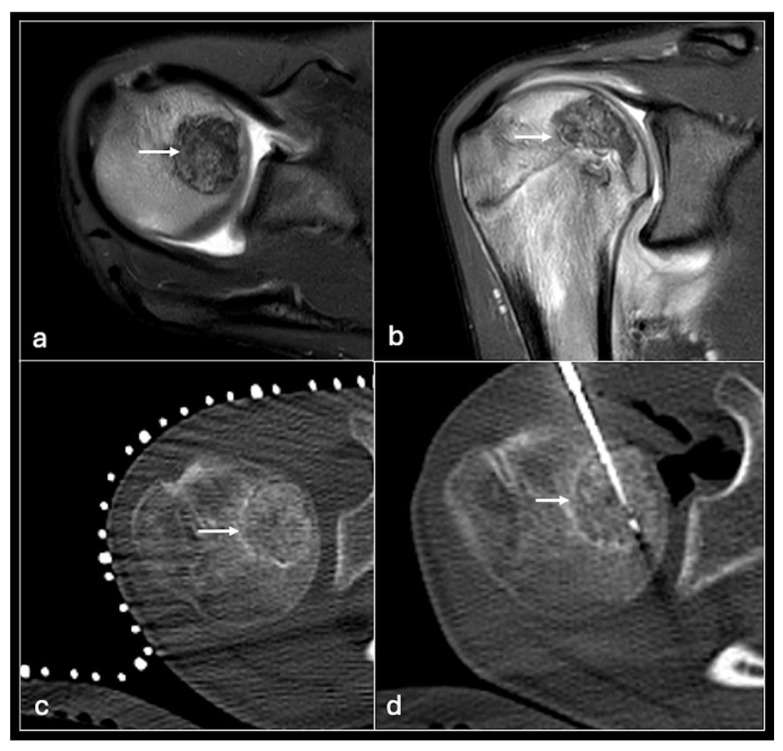
STIR axial (**a**), coronal (**b**) showing chondroblastoma in the humeral head (arrow) treated with CT-guided cryotherapy (**c**,**d**).

Cryoablation is the direct rapid cooling of lesions to temperatures as low as −100 °C to induce crystallisation of intracellular water thus causing apoptosis. This is achieved using rapidly expanding gas (usually argon) to cause a rapid drop in temperature via a phenomenon termed the Joule–Thompson effect [50]. Multiple freeze–thaw cycles are usually required with the latter achieved using helium gas infusion (Figure 12).

Indications are more wide-ranging than other forms of ablation. Curative oncological indications include benign desmoid tumours, osteoid osteomas, vascular malformations, aneurysmal bone cysts and neuromas [50,51]. Malignant lesions such as bone metastases can be treated curatively but palliation of painful bone metastases is a more established process [52,53,54].

Like other thermoablation methods, cryoablation is achieved by percutaneously inserting probes into the lesion. Unlike RFA, multiple needles can be used allowing more flexibility in tumours of different geometry and coverage of a larger ablation area. However, the ablation process takes longer and is more expensive than other options. As well as general thermoablation risks to the skin and perilesional structures, specific risks include cryomyositis (peritumoural muscle inflammation) and cryoshock (a systemic inflammatory response from the release of inflammatory mediators). Numerous methods can be employed to minimise inadvertent freezing risk such as hydrodissection and active or passive warming of the adjacent structures [33,55]. Temperature monitoring and direct visualisation of the ice ball using CT or ultrasound during cryotherapy can also reduce the risk of injuring adjacent structures [56,57].

• High-Frequency Ultrasound

Magnetic Resonance-Guided High-Intensity Focused Ultrasound (MRg-HIFU) is the non-invasive focusing of ultrasound onto a lesion to induce heat and coagulation necrosis within the lesion. Musculoskeletal applications include osteoid osteoma/osteoblastoma and bone metastases (particularly for pain relief) but clinical utility specifically in the cure of musculoskeletal tumours remains relatively sparse [58,59,60].

The key benefit of percutaneous ablation is reduced complications from the non-invasive nature such as a much lower risk of fracture or infection. However, efficacy is limited to relatively small superficial structures within thin, flat bones and away from any perilesional vital structures [58,60].

#### 3.5.2. Cement Augmentation

**Figure 13:**
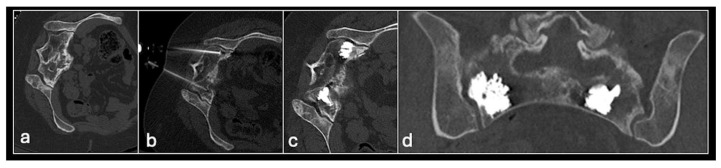
Axial CT (**a**) showing metastasis in the sacrum. Images (**b**–**d**) showing sacroplasty with cement in both sacral ala.

**Figure 14:**
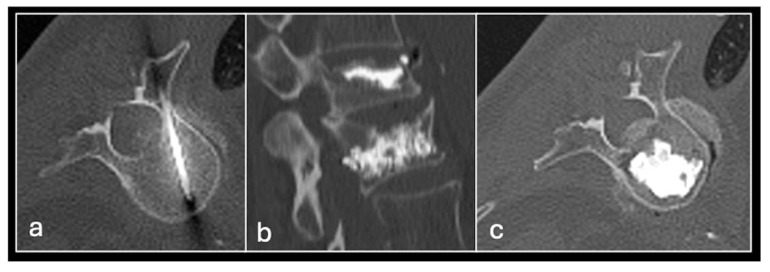
CT-guided vertebroplasty showing needle in the vertebral body (**a**) and sagittal (**b**) and axial (**c**) showing cement within the vertebral bodies.

Cement augmentation is the injection of polymethylmethacrylate cement (PMMA) mixture into a bony defect to restore bony structural integrity and provide pain relief. The most common form is vertebroplasty in the context of vertebral compression fractures which—in the setting of oncology—include pathological fractures with underlying myeloma or metastases with resultant pain and disability refractory to conservative treatment. The procedure is also a useful adjunct following percutaneous ablation to restore structural integrity following bone destruction, particularly for larger lesions [33] (Figure 13 and Figure 14).

A relatively novel procedure described in the literature combines Ablation, Osteoplasty, Reinforcement, and Internal Fixation (AORIF) and it has been applied most commonly in the pelvis and proximal femora [60,61,62]. This is performed by introducing cannulated screws into osteolytic lesions as portals for ablation, balloon osteoplasty, and the delivery of bone cement to facilitate the treatment of these lesions.

The most common type of cement augmentation at our centre is vertebroplasty. This is typically performed via a transpedicular approach into the lesion using an 11 G or 14 G bone biopsy needle system. Biopsy samples may be taken with the PMMA and then injected into the defect. A modified procedure is the kyphoplasty in which cement injection is preceded by the inflation of a balloon device in the vertebral body to create space with the cavity then filled with the cement. This is more effective in pain relief and minimises leakage but results in a longer and more expensive procedure [4,63].

Other variants with similar underlying principles include sacroplasty and acetabuloplasty. Complications to be aware of include cement leakage, which can lead to cement thrombosis, pulmonary emboli and compression of adjacent structures [64,65]. Nevertheless, cement augmentation generally remains a relatively safe and effective minimally invasive procedure, which can greatly improve patient quality of life in a palliative setting.

## 4. Conclusions

Advancements in musculoskeletal interventional radiology have yielded a wide range of diagnostic and therapeutic minimally invasive procedures. We illustrated how these have introduced more flexibility to the management of soft tissue and bone tumours and provide alternatives to more traditional surgical and oncological approaches in curative and palliative settings.

It is virtually certain that the continued development of minimally invasive tissue sampling, ablation, and augmentation procedures amongst others will add to the arsenal of treatment options across a range of clinical oncological applications. This study has provided the reader with insight into how these techniques and principles can be applied to different clinical scenarios to optimise patient management.

## Figures and Tables

**Table 1:** Table describing the major advantages and disadvantages of different methods of ablation techniques in musculoskeletal IR.

	Mechanism	Advantages	Disadvantages
**Chemical ablation**	Injection of cytotoxic sclerosant material into a cavity	• Relatively minimal complication rates compared to thermoablation	• Often requieres multiple procedures to complete treatment • Largely limited to treatment of cystic lesions • Sclerosant leakage can cause embolism and tissue necrosis
**Radiofrequency Ablation (RFA)**	Delivery of alternating radiofrequency current to induce cytotoxic heat	• Relatively quick • Improved bone consolidation compared to other types of ablation (reduced fracture risk) • Normal cells are relatively resistant to heat shock	• Transient pain is more common and severe • Skin burn which can be severe
**Microwave Ablation (MWA)**	Delivery of alternating radiofrequency current to induce heat and cause tumour death	• Fastest at inducing effective coagulation • Can extend deeper with reduced inherent resistance	• Further research needed into optimising parameters
**Interstitial Laser Ablation (ILA)**	Laser light- induced heating to causes tumour cell death	• Very precise • Safe and does not require thermoprotection • No risk of skin burn or frostbite	• Limited to benign and small tumours
**Cryoablation**	Application of freeze-–thaw cycles to induce tumour cell death	• More effective for sclerotic lesions as it can better overcome the thermal insulation properties of cortical bone • Less painful thanks to cryoanalgesia • Cryoadhesional retraction can be used to manipulate the targeted structures • MRI can accurately monitor the cryoablation zone	• Frostbite • Cryoshock • Cryomyositis • Relatively increased bleeding risk compared to heat-based methods as there is no coagulative phase in CA • Limited use in larger tumours (i.e., ≥3 cm or if ≥3 tumours) • Relatively expensive, particularly if using MRI-compatible equipment
**High- Frequency Ultrasound (HIFU)**	Cytotoxic heat is generated by focusing ultrasound waves from multiple sources to a common point	• Noninvasive • Relatively safe with minimal risks of burn and inadvertent neurovascular injury	• Can only be used for very superficial lesions in soft tissue or bones with thin cortices • Must be combined with stabilisation if in weight-bearing bones • Works poorly with thermoprotection techniques

## Data Availability

No new data were created or analysed in this study. Data sharing is not applicable to this article.
